# Implementation of a Core–Shell Design Approach for Constructing MOFs for CO_2_ Capture

**DOI:** 10.1021/acsami.3c03457

**Published:** 2023-05-04

**Authors:** Yiwen He, Paul Boone, Austin R. Lieber, Zi Tong, Prasenjit Das, Katherine M. Hornbostel, Christopher E. Wilmer, Nathaniel L. Rosi

**Affiliations:** † Department of Chemistry, University of Pittsburgh, Pittsburgh, Pennsylvania 15260, United States; ‡ Department of Chemical and Petroleum Engineering, University of Pittsburgh, 3700 O’Hara Street, Pittsburgh, Pennsylvania 15261, United States; § Department of Mechanical Engineering & Materials Science, University of Pittsburgh, 3700 O’Hara Street, Pittsburgh, Pennsylvania 15261, United States; ∥ Department of Electrical and Computer Engineering, University of Pittsburgh, 3700 O’Hara Street, Pittsburgh, Pennsylvania 15261, United States; ⊥ Clinical and Translational Science Institute, University of Pittsburgh, Meyran Avenue, Suite 7057, Pittsburgh, Pennsylvania 15213, United States

**Keywords:** metal−organic frameworks, porous materials, carbon capture, gas adsorption, DAC

## Abstract

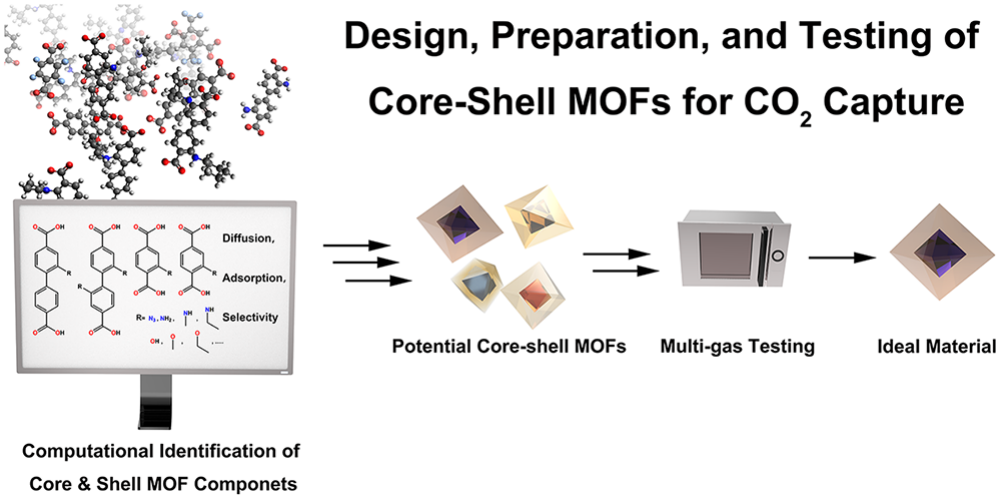

Adsorption-based capture of CO_2_ from flue gas and from air requires materials that have a high affinity for CO_2_ and can resist water molecules that competitively bind to adsorption sites. Here, we present a core–shell metal–organic framework (MOF) design strategy where the core MOF is designed to selectively adsorb CO_2_, and the shell MOF is designed to block H_2_O diffusion into the core. To implement and test this strategy, we used the zirconium (Zr)-based UiO MOF platform because of its relative structural rigidity and chemical stability. Previously reported computational screening results were used to select optimal core and shell MOF compositions from a basis set of possible building blocks, and the target core–shell MOFs were prepared. Their compositions and structures were characterized using scanning electron microscopy, transmission electron microscopy, and powder X-ray diffraction. Multigas (CO_2_, N_2_, and H_2_O) sorption data were collected both for the core–shell MOFs and for the core and shell MOFs individually. These data were compared to determine whether the core–shell MOF architecture improved the CO_2_ capture performance under humid conditions. The combination of experimental and computational results demonstrated that adding a shell layer with high CO_2_/H_2_O diffusion selectivity can significantly reduce the effect of water on CO_2_ uptake.

## Introduction

Increasing carbon dioxide (CO_2_) in the atmosphere is primarily responsible for the greenhouse effect and global warming.^1−4^ According to NOAA Earth System Research Laboratories (ESRL), the global monthly mean CO_2_ has increased by about 23% since 1980. High levels of CO_2_ emissions contribute to an average annual temperature increase of 1.5 °C compared to the preindustrial level.^5^ Effective methods are required to minimize CO_2_ emissions and decrease CO_2_ levels in the atmosphere.

Removing CO_2_ from combustion emissions or directly from the atmosphere requires materials that are highly selective at capturing CO_2_ over other gas molecules that can competitively interact with the material.^6−10^ Various methods for CO_2_ capture have been developed, including adsorption and membrane-based separations.^11−13^ Amine-based sorbents, both liquid and solid, have been extensively studied.^14−18^ While these materials can perform up to 98% CO_2_ capture,^14^ their performance often suffers in the presence of water vapor, which competitively binds to adsorption sites.^12^ Therefore, the goal of selective CO_2_ capture presents a significant material design challenge: an ideal target material would have a high affinity to CO_2_ while limiting competitive adsorption of H_2_O.^19−23^ Metal–organic frameworks (MOFs) have proven useful for CO_2_ sorption and separation processes,^24−29^ and with their chemical and structural tunability, they may be ideal material platforms for addressing this challenge.

**Figure 1:**
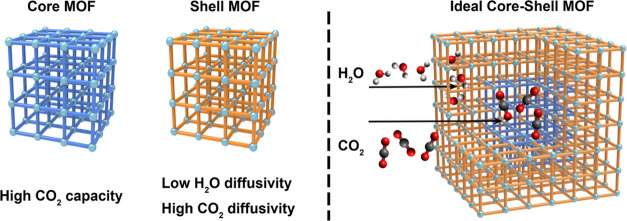
Design of an optimal core–shell MOF for CO_2_ capture.

We recently reported a material design approach based on multicomponent core–shell metal–organic frameworks (MOFs)^30−39^ in which the core and shell domains were computationally selected to perform particular functions.^40^ This type of hybrid design can enable superior properties compared to a single-component MOF because each MOF layer can be optimized separately.^33,41,42^ Specifically, we used this approach to identify potential core MOFs with high CO_2_ capacity and shell MOFs with high CO_2_ diffusivity over H_2_O in order to create a material where CO_2_ adsorption sites could saturate with CO_2_ even in the presence of competitively adsorbing water vapor (Figure 1). In this study, we take the first steps toward validating this design approach by preparing target core–shell MOFs and experimentally testing their competitive adsorption properties in mixed gas streams that approximate flue gas. We demonstrate that specific multidomain core–shell MOFs outperform the individual single-domain MOFs by reducing the effect of humidity on CO_2_ uptake. We also show that the sequence of domains within the core–shell MOF is critical for achieving the desired performance.

## Results and Discussion

### MOF Selection, Synthesis, and Characterization

**Figure 2:**
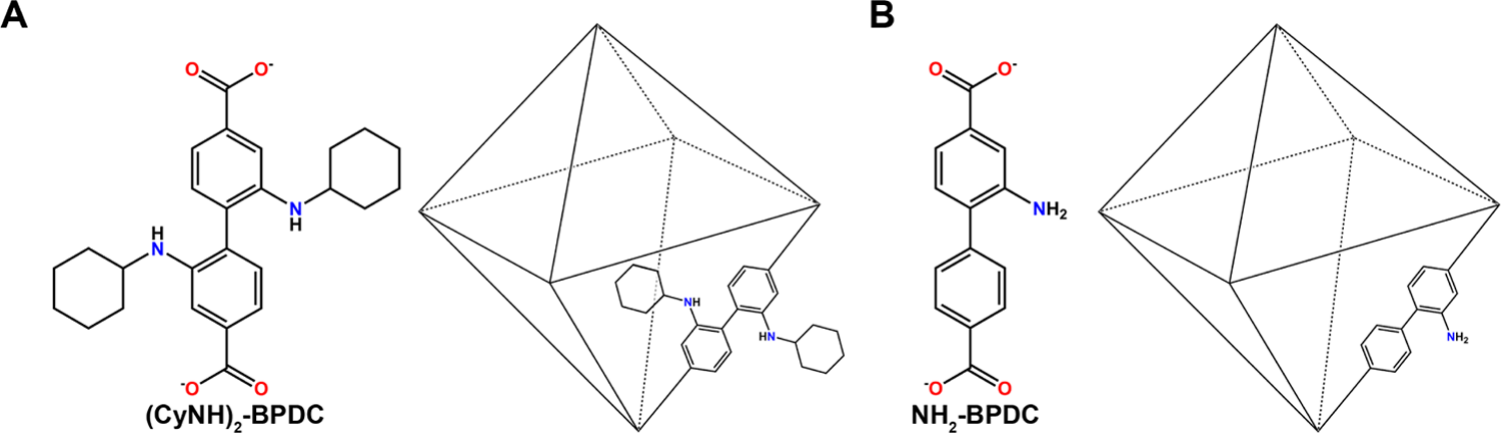
Core ligand (A) and shell ligand (B) for potential optimal core–shell MOF.

We chose zirconium (Zr)-based UiO-66/67 MOFs for our studies because (i) they are structurally robust; (ii) isoreticular families can be prepared using a wide variety of dicarboxylate ligands;^43^ and (iii) previous studies from our group demonstrated the successful synthesis of core–shell MOFs using UiO-67.^37^ For both UiO-66 and -67, a total of 28 terephthalate (BDC) and 28 biphenyl-4,4′-dicarboxylate (BPDC) derivatives (Figure S1), respectively, were proposed as potential ligands for the core and shell domains. Since it was practically impossible to synthesize and test all of the different core–shell MOF combinations (there are 1512 total possibilities with compositionally distinct core and shell domains), computational screening of these different MOFs was performed to determine adsorption and diffusivity of N_2_, CO_2_, and H_2_O at conditions relevant for DAC.^40^ From these data, we identified potential shell MOFs that would allow rapid diffusion of CO_2_ and slow diffusion of H_2_O, reasoning that such MOFs would limit H_2_O penetration to the core MOF. A UiO-67 derivative containing 2-amino-[1,1′-biphenyl]-4,4′-dicarboxylate (Figure 2B), NH_2_-BPDC, was selected for the shell because it had the highest CO_2_ diffusivity of 49.8 m^2^/s of the MOFs screened and a high CO_2_/H_2_O diffusion selectivity of 307. Potential core MOFs were those predicted to selectively capture CO_2_ over N_2_ and H_2_O. The UiO-67 derivative containing 2,2′-diclyclohexylamino-[1,1′-biphenyl]-4,4′-dicarboxylate (Figure 2A), (CyNH)_2_-BPDC, was chosen because it displayed the highest CO_2_/N_2_ adsorption selectivity (31) and a high CO_2_ capacity of 0.0104 cm^3^/g. Although this prior work focused on DAC conditions (e.g., 400 ppm CO_2_), the MOFs with the highest predicted CO_2_ adsorption under very dilute conditions are still going to be the highest at a 15% CO_2_ concentration. Similarly, the water diffusivity simulation results from a prior work are also transferable to the present study.

**Figure 3:**
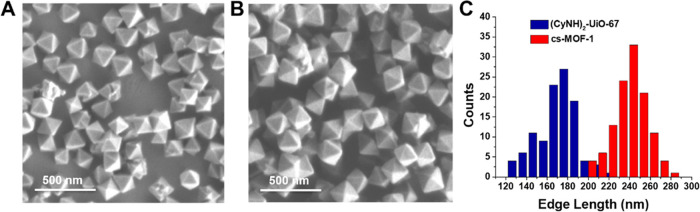
(A) SEM image of (CyNH)_2_-UiO-67. (B) SEM image of cs-MOF-1. (C) Size distribution of (CyNH)_2_-UiO-67 and cs-MOF-1 (based on 100 counts).

To validate our computational predictions, we prepared and tested the individual target core and shell MOFs (i.e., (CyNH)_2_-UiO-67 and NH_2_-UiO-67) as well as the target core–shell MOF, (CyNH)_2_-UiO-67⊂NH_2_-UiO-67, hereafter referred to as cs-MOF-1 (core–shell MOF-1). In addition, we prepared and tested the inverse of cs-MOF-1, NH_2_-UiO-67⊂(CyNH)_2_-UiO-67 (cs-MOF-2), to determine the importance of the core/shell sequence in determining cs-MOF properties. All MOFs were synthesized using established methods,^37^ and detailed synthetic protocols and characterization data are included in the Supporting Information (Sections 3 and 4). For cs-MOF-1, (CyNH)_2_-UiO-67 crystal seeds were first synthesized, washed with dry *N*,*N*-dimethylformamide (DMF), and then placed in a shell growth solution containing zirconium (IV) chloride (ZrCl_4_), H_2_-NH_2_-BPDC, acetic acid, and DMF. The mixture was heated at 65 °C for 40 h. The resulting cs-MOF-1 crystals were collected and washed prior to characterization. Powder X-ray diffraction (PXRD) was used to verify that cs-MOF-1 was isostructural to UiO-67 (Figure S8). Scanning electron microscopy (SEM) imaging was used to determine the MOF particle size for both the seed (CyNH)_2_-UiO-67 crystals and cs-MOF-1 (Figure 3A,B). The size distribution increased from 174 ± 36 nm for the seeds to 249 ± 37 nm for cs-MOF-1 (Figure 3C), consistent with the growth of a NH_2_-UiO-67 shell on the (CyNH)_2_-UiO-67 seeds.

**Figure 4:**
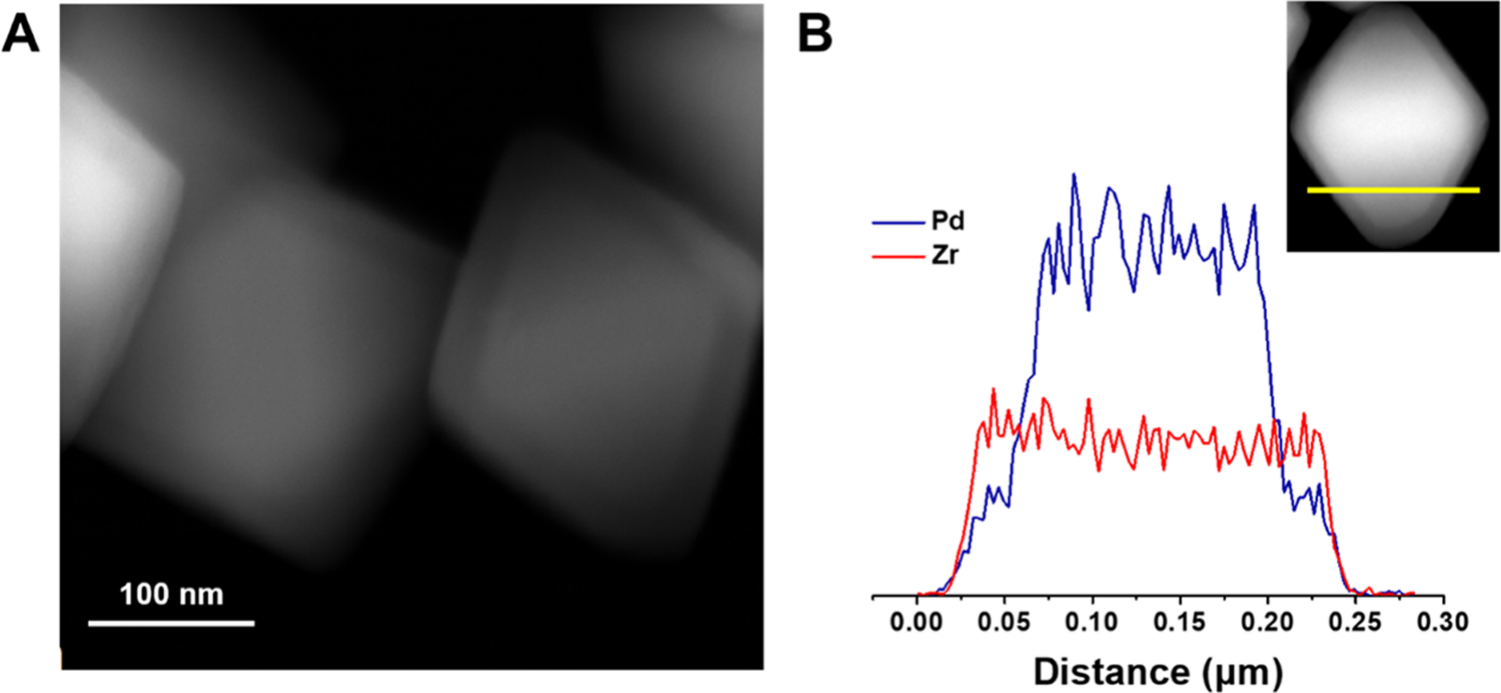
(A) STEM image of (CyNH)_2_-UiO-67⊂UiO-67. (B) STEM-EDS line-scan data of (CyNH)_2_-UiO-67⊂UiO-67.

During shell growth, linker exchange can occur between linkers in the shell growth solution and linkers in the core MOF.^37^ At the extreme of linker exchange, it may be possible to form a mixed-ligand multivariate MOF with a 50:50 ratio of ligands instead of a core–shell MOF. In order to clearly distinguish the core and shell domains microscopically and to verify their chemical constitution, we applied a palladium “staining” approach used in our previous study,^37^ where palladium coordinates to a specific MOF ligand and can then be used to identify the location of that ligand via energy-dispersive X-ray spectroscopy (EDS). To assess the viability of this approach, we first soaked both NH_2_-UiO-67 and (CyNH)_2_-UiO-67 in bis(acetonitrile)dichloropalladium(II), washed thoroughly, and then imaged and analyzed the crystals with scanning transmission electron microscopy-EDS (STEM-EDS) to determine the presence of Pd. Due to the presence of the Lewis basic amino functional groups, both NH_2_-UiO-67 and (CyNH)_2_-UiO-67 (Figures S11–S14) coordinated to Pd(II). Therefore, this method would not allow us to distinguish the core and shell domains. We decided instead to prepare (CyNH)_2_-UiO-67⊂UiO-67 because UiO-67, which contains biphenyl-4,4′-dicarboxylate (BPDC) linkers, and NH_2_-UiO-67 are expected to behave similarly in the context of synthesizing core–shell MOFs. In fact, we would expect more linker exchange to occur when using UiO-67 as a shell compared to NH_2_-UiO-67 because the BPDC linkers have less steric bulk than NH_2_-BPDC. In a previous work, we demonstrated that linker exchange could be limited by increasing the linker steric bulk.^37^ Therefore, if core and shell ligand domains could be clearly observed for (CyNH)_2_-UiO-67⊂UiO-67, we would expect cs-MOF-1 to have similarly distinct core and shell domains. First, we proved that Pd does not associate with UiO-67 when the material is soaked in bis(acetonitrile)dichloropalladium(II) (Figures S19 and S20). (CyNH)_2_-UiO-67⊂UiO-67 was then prepared, washed thoroughly with acetonitrile (ACN), and then soaked overnight in an ACN solution of bis(acetonitrile)dichloropalladium(II). STEM images (Figure 4A) revealed a core–shell structure, with the lighter core indicating the presence of Pd. STEM-EDS was then used to collect line-scanning spectra (Figure 4B) of Zr and Pd. The Pd signal was predominantly detected at the “core”, while the Zr signal was detected throughout the whole “core–shell” crystal. A weak signal from Pd also appeared within the shell layer, which can be attributed to a small amount of linker exchange during the shell growth process. However, the lower signal intensity of Pd in the shell layer indicates that the shell is predominantly UiO-67. Collectively, these data confirm the 3-D architecture and division of core and shell domains in the core–shell MOF.

### Single-Gas Adsorption Studies

We proceeded to examine the gas sorption properties of the individual core and shell MOFs, as well as cs-MOF-1 and cs-MOF-2. N_2_ sorption isotherms collected at 77 K for each MOF (Figure S21) confirm microporosity. From these data, we calculated a Brunauer–Emmett–Teller (BET) surface area (SA) of 2280 m^2^ g^–1^ for NH_2_-UiO-67 and 1780 m^2^ g^–1^ for (CyNH)_2_-UiO-67. The lower BET SA for (CyNH)_2_-UiO-67 is attributed to the bulkier functional groups. The BET SAs for cs-MOF-1 and cs-MOF-2 were 1830 and 1810 m^2^ g^–1^, respectively, which lie between those of NH_2_-UiO-67 and (CyNH)_2_-UiO-67, as expected due to their mixed ligand composition. CO_2_ and N_2_ adsorption isotherms were also collected at 298 K (Figures S22 and S23). The amounts of CO_2_ and N_2_ adsorbed for cs-MOF-1 and cs-MOF-2 lie between NH_2_-UiO-67 and (CyNH)_2_-UiO-67 under all pressures. The CO_2_ capacity for (CyNH)_2_-UiO-67 is higher than that for NH_2_-UiO-67, which is consistent with our computational predictions.^40^

### Multigas Adsorption Studies

**Figure 5:**
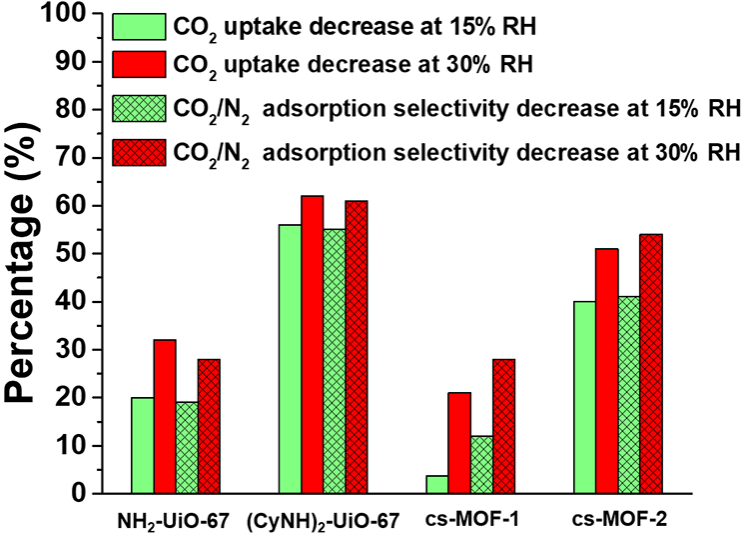
Comparison of multigas sorption results. The plot displays the percent decrease in CO_2_ uptake and CO_2_/N_2_ selectivity at different RH compared to the 0% RH condition.

To evaluate the CO_2_ capture performance of each MOF under multigas and humid conditions, we constructed a multigas manifold and sample holder for measuring the gas uptake using flow controllers and a gas chromatograph (GC) with a thermal conductivity detector (Scheme S1).^44^ Approximately 100 mg of MOF sample was loaded into the sample holder, which was then evacuated overnight in a 120 °C vacuum oven. The loaded sample holder was connected to the multigas manifold, and a certain ratio of N_2_/CO_2_/H_2_O was allowed to flow through the sample. After reaching equilibrium, the MOF sample was degassed at 120 °C with He flow and GC was used to determine the composition of the effluent. A 15:85 CO_2_/N_2_ gas mixture, an approximation of the flue gas composition, was used for our initial tests of NH_2_-UiO-67, (CyNH)_2_-UiO-67, cs-MOF-1, and cs-MOF-2. Three different RH values (0, 15, and 30%) were used to determine the material performance and stability under humid conditions and the potential benefits of using a core–shell MOF design. Two trials were performed at each condition to confirm repeatability, and the data are summarized in Tables S1–S3. To most effectively present these data, we compared the CO_2_ uptake and CO_2_/N_2_ selectivity for each MOF at each RH condition to determine how humidity affects performance, determining specifically the percent decrease in uptake and selectivity relative to the 0% RH condition (Figure 5).

Compared to their performance at 0% RH, NH_2_-UiO-67 and (CyNH)_2_-UiO-67 showed 20 and 56% decreases in CO_2_ uptake, respectively, under 15% RH and 32 and 62% decreases, respectively, under 30% RH (Figure 5 and Tables S2 and S3). A similar decrease in CO_2_/N_2_ adsorption selectivity was observed for these materials under the same conditions. Reduced CO_2_ uptake in the presence of water vapor is common,^44,45^ and this reduction can be attributed to competitive adsorption between CO_2_ and H_2_O, which can both hydrogen bond with the amino groups. We next tested cs-MOF-1 to determine if the core–shell architecture affects the CO_2_ capture performance. The decreases in CO_2_ uptake at 15% RH and 30% RH are 3.7 and 21% (Figure 5 and Tables S2 and S3), respectively, a significant improvement over both individual MOFs. Based on our simulation results,^40^ NH_2_-UiO-67 has a higher CO_2_/H_2_O diffusion selectivity. With NH_2_-UiO-67 as the shell, diffusion of H_2_O is limited, while CO_2_ can diffuse to the core and occupy the core adsorption sites. The core–shell design therefore mitigates the effect of humidity on (CyNH)_2_-UiO-67 CO_2_ uptake. The decrease in CO_2_/N_2_ selectivity for cs-MOF-1 is slightly smaller than NH_2_-UiO-67 yet significantly smaller compared to (CyNH)_2_-UiO-67, again indicating that that the core–shell MOF architecture confers distinct advantages over the individual MOFs alone. We note that the CO_2_ uptake and CO_2_/N_2_ adsorption selectivity for cs-MOF-1 under all RH conditions were lower than those of (CyNH)_2_-UiO-67, which can be attributed to the fact that cs-MOF-1 is partially composed of NH_2_-UiO-67, which has a lower CO_2_ uptake than (CyNH)_2_-UiO-67. Therefore, although NH_2_-UiO-67 can mitigate the effect of humidity on (CyNH)_2_-UiO-67, it would be expected to decrease the CO_2_ uptake of cs-MOF-1 relative to (CyNH)_2_-UiO-67 alone. Further studies on controlling core and shell thicknesses could be performed to most effectively balance the shell’s role in mitigating the negative effect of humidity while also minimizing its effect on the overall CO_2_ capture performance. We also studied cs-MOF-2 to determine how the core–shell MOF sequence influences performance. In this case, the percent decreases in CO_2_ uptake and CO_2_/N_2_ adsorption selectivity at 15 and 30% RH relative to 0% RH lie between the observed decreases for the single-component MOFs (Figure 5 and Tables S2 and S3). Collectively, we can conclude from these data that a shell layer with high CO_2_/H_2_O diffusion selectivity can reduce the detrimental effects of humidity on the CO_2_ capture performance of the core MOF.

Water vapor can cause hydrolytic MOF decomposition, which can significantly affect the gas sorption properties.^46^ After each multigas sorption test, PXRD (Figures S24–S27) and N_2_ adsorption (Figures S28–S31) at 77 K were performed to assess the stability of the MOFs under humid conditions. The PXRD patterns indicate that all four MOFs tested maintain their crystallinity after the multigas sorption tests; however, after exposure to 30% RH NH_2_-UiO-67 and (CyNH)_2_-UiO-67, both show a significantly decreased peak intensity. The N_2_ adsorption tests at 77 K revealed a decrease in BET SA for each MOF studied after the multigas sorption tests under humid conditions. The BET SA decreases after the 15% RH tests were 5.7% for NH_2_-UiO-67, 7.3% for (CyNH)_2_-UiO-67, 0.5% for cs-MOF-1, and 4.4% for cs-MOF-2. These numbers increased to 21, 22, 12, and 18% after 30% RH tests for NH_2_-UiO-67, (CyNH)_2_-UiO-67, cs-MOF-1, and cs-MOF-2, respectively. These results indicate that some of the MOFs were partially decomposed under humid conditions, which is widely observed for UiO MOFs,^46^ and higher RH (30%) affects the MOF structure more than the low RH (15%) condition. We note that the observed lower CO_2_ capture performance under 30% RH conditions could be attributed to humidity-induced structural degradation.

## Conclusions

In summary, we tested and validated a core–shell design approach for identifying MOFs for carbon capture under humid conditions. cs-MOF-1 was selected via computational screening,^40^ and its carbon capture performance was tested and compared to the individual core and shell MOF components. Our data indicate that coating a MOF with a high capacity and selectivity for CO_2_ ((CyNH)_2_-UiO-67) with a protective MOF shell having a high CO_2_/H_2_O diffusion selectivity (NH_2_-UiO-67) effectively mitigates the negative consequences of competitive water adsorption. While we emphasize that further improvements to MOF stability under humid conditions as well as improved CO_2_ capacity and selectivity would be required for real-world applications, this demonstration represents an important first step toward realizing how MOF stratification can significantly improve CO_2_ capture performance in humid conditions.

## Supporting Information Available

Syntheses and experimental protocols of ligands and MOFs, characterizations of MOFs including PXRD data, microscopic data, gas sorption data, setup, and testing results of multigas sorption (PDF)
