# Supplementary material for: Implementation of a Core–Shell Design Approach for Constructing MOFs for CO2 Capture
Source: ACS Appl Mater Interfaces. 2023 May 4;15(19):23337–42. doi: 10.1021/acsami.3c03457 (PMC10197066; doi:10.1021/acsami.3c03457)
Supplement: Supplementary file 1 — am3c03457_si_001.pdf [file am3c03457_si_001.pdf]

Supporting information

Implementation of a Core-shell Design Approach for Constructing MOFs for CO_2 Capture

Yiwen He[a], Paul Boone[b], Austin R. Lieber[c], Zi Tong[a], Prasenjit Das[a], Katherine M. Hornbostel[bc], Christopher E. Wilmer[bde], Nathaniel L. Rosi[ab]*

**Corresponding author: nrosi@pitt.edu*

[a]Department of Chemistry, University of Pittsburgh, Pittsburgh, Pennsylvania, 15260.

[b]Department of Chemical and Petroleum Engineering, University of Pittsburgh, 3700 O'Hara Street, Pittsburgh, Pennsylvania 15261.

[c]Department of Mechanical Engineering & Materials Science, University of Pittsburgh, 3700 O'Hara Street, Pittsburgh, Pennsylvania 15261.

[d]Department of Electrical and Computer Engineering, University of Pittsburgh, 3700 O'Hara Street, Pittsburgh, Pennsylvania 15261

[e] Clinical and Translational Science Institute, University of Pittsburgh, Meyran Ave Suite 7057, Pittsburgh, PA 15213

Table of Contents

1. General Methods 4

2. Proposed ligands for computational MOF screening 5

3. Synthesis and characterization of MOF ligands 6

3.1. Dimethyl 2-nitro-1,1'-biphenyl-4,4'-dicarboxylate (1) 6

3.2. Dimethyl 2-amino-1,1'-biphenyl-4,4'-dicarboxylate (2) 6

3.3. 2-amino-1,1'-biphenyl-4,4'-dicarboxylic acid (3) 6

3.4. Dimethyl-2,2'-dinitro-[1,1'-biphenyl]-4,4'-dicarboxylate (1’) 7

3.5. Dimethyl 2,2'-diamino-[1,1'-biphenyl]-4,4'-dicarboxylate (2’) 7

3.6. 2,2'-diamino-[1,1'-biphenyl]-4,4'-dicarboxylic acid (3’) 7

3.7. 2,2'-dicyclohexylamino-[1,1'-biphenyl]-4,4'-dicarboxylic acid (4’) 7

4. Syntheses and characterization of MOFs 9

4.1. NH_2-UiO-67 9

4.2. $(CyNH)_2$-UiO-67 10

4.3. $(CyNH)_2$-UiO-67⊂NH_2-UiO-67(cs-MOF-1) 11

4.4. NH_2-UiO-67⊂$(CyNH)_2$-UiO-67 (cs-MOF-2) 15

4.5. $(CyNH)_2$-UiO-67⊂UiO-67 16

5. Single-gas adsorption isotherms 18

5.1. N_2 adsorption isotherms at 77 K 18

5.2. CO_2 adsorption isotherms at 298 K 18

5.3. N_2 adsorption isotherms at 298 K 19

6. Multi-gas testing 20

6.1. System design 20

6.2. Multi-gas tests results 21

6.3. Characterization of MOF stability after Multi-gas Tests 22

7. References 26

1. General Methods

All reagents and solvents were commercially available and used as received.

^1H NMR spectra were obtained using Bruker Avance III 300/400/500 MHz spectrometers. Chemical shifts are in parts per million (ppm) using the residual solvent peak ($CDCl_3$, DMSO-d6, or D_2O) as reference.

Powder X-ray diffraction (PXRD) patterns were collected using a Bruker AXS D8 Discover powder diffractometer at 40 kV, 40 mA for Cu Kα, (λ = 1.5406 Å) with a scan speed of 0.20 s/step from 5 to 30° at a step size of 0.02°. The data were analyzed using the EVA program from the Bruker powder analysis software package. The simulated powder patterns were calculated using Mercury 3.8 based on MOF crystal structures.

Scanning electron microscopy (SEM) data were collected using a ZEISS Sigma 500 VP scanning electron microscope. Samples were dispersed in ethanol and drop cast on TEM grids (Ted Pella Inc., 200 mesh carbon film copper grids, catalog No. NC0733370). The TEM grids were dried under ambient conditions before SEM studies. A STEM sample holder was used to mount the TEM grids.

Transmission electron microscopy (TEM) images used to determine size distributions of MOF crystallites were collected on an FEI Morgagni 268 operated at 80 kV with an AMT side mount CCD camera system. Scanning transmission electron microscopy - energy dispersive X-ray spectroscopy (STEM) imaging and STEM-EDS studies were conducted on a JEOL JEM-2100F equipped with a Gatan Orius camera operated at 200 kV. Samples were dispersed in acetonitrile and drop cast on TEM grids [Ted Pella Inc., 200 mesh carbon film copper grids, catalog no. NC0733370]. The TEM grids were dried under ambient conditions before TEM and STEM-EDS analyses. EDS data were acquired using 1024 channels from 0 to 20 keV. Elemental maps were collected for 10–15 min with a pixel dwell time of 100 μs and a pixel resolution of 1024 × 1024. EDS maps and line-scans for zirconium and palladium were generated using the Zr Kα1 line intensity at 15.7 keV and the Pd Lα1 line intensity at 2.8 keV.

Gas adsorption isotherms were collected on a Quantachrome Autosorb-1 instrument or on a Micromeritics 3-flex gas adsorption analyzer. Approximately 40-60 mg of each sample was exchanged with dichloromethane 3 times a day for 1 day at 65 ºC to remove N,N-dimethylformamide. After that, samples were degassed at 100 ºC for 24 h on a Micromeritics SmartVacPrep under vacuum. A liquid N_2 bath was used for the N_2 adsorption experiments at 77 K. A water bath was used for N_2 and CO_2 adsorption experiment at 298 K. Ultra-high purity grade N_2 and CO_2 (99.999 %) was used.

2. Proposed ligands for computational MOF screening

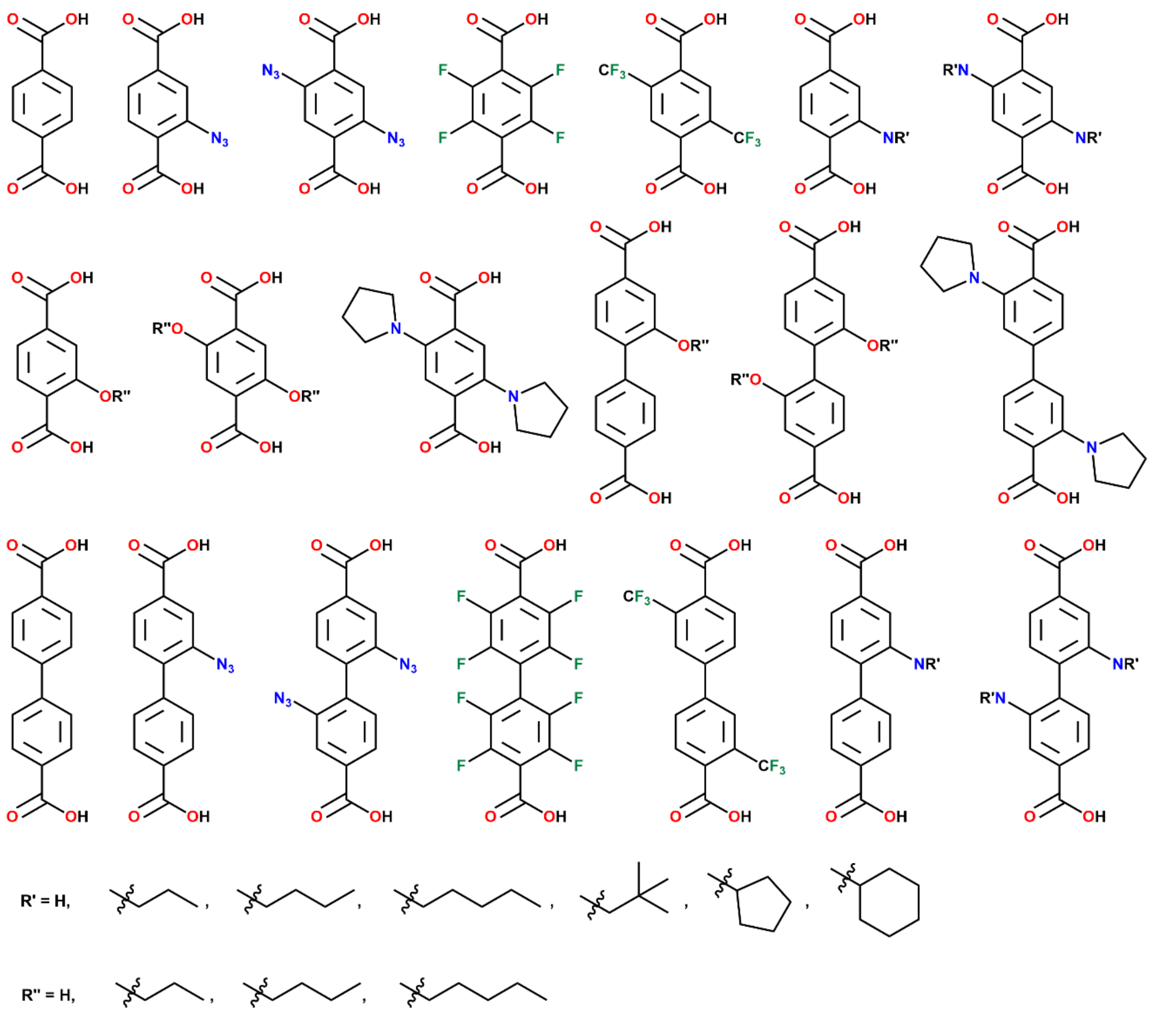

Figure S1. Proposed ligands for computational MOF screening.

3. Synthesis and characterization of MOF ligands

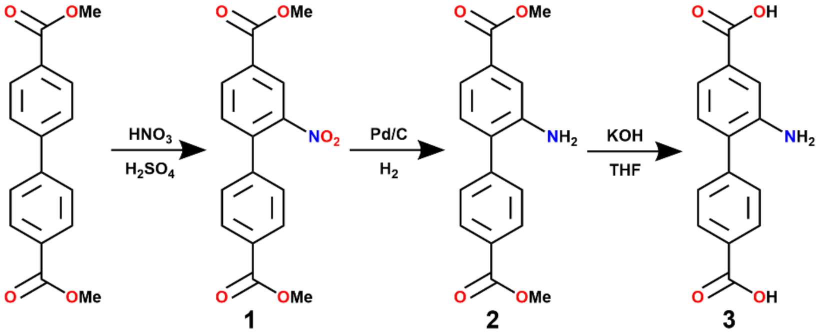

Figure S2. Synthesis of 2-amino-1,1'-biphenyl-4,4'-dicarboxylic acid.

3.1. Dimethyl 2-nitro-1,1'-biphenyl-4,4'-dicarboxylate (1)

Compound **1** was synthesized according to literature conditions.[1]

3.2. Dimethyl 2-amino-1,1'-biphenyl-4,4'-dicarboxylate (2)

To a 100 mL Schlenk flask equipped with a stir bar, were added compound **1** (710 mg, 2 mmol), 10 wt. % palladium on carbon (70 mg) and ethyl acetate (30 mL). The Schlenk flask was quickly evacuated on a Schlenk line and then backfilled with N_2 gas. This evacuation and backfill process was repeated 3 times. The Schlenk flask was then evacuated and attached to a H_2 balloon. The reaction mixture was stirred at room temperature under H_2 atmosphere and monitored via thin layer chromatography (TLC). After 6 hours, the reaction was stopped by removing the Pd catalyst via vacuum filtration through a celite cake. The filtrate was concentrated *in vacuo* to yield a light-yellow solid (compound 2) (570 mg, 93%). Compound 2 was used without further purification. ^{1}H NMR (400 MHz, $CDCl_3$) δ 8.13 (m, 2H), 7.55 (m, 2H), 7.48 (dd, J = 7.9, 1.4 Hz, 1H), 7.45 (d, J = 1.4 Hz, 1H), 7.18 (d, J = 7.9 Hz, 1H), 3.95 (s, 3H), 3.92 (s, 3H), 3.86 (s, 2H).

3.3. 2-amino-1,1'-biphenyl-4,4'-dicarboxylic acid (3)

Compound **3** was synthesized using compound 2 as the starting material based on literature conditions.[1] ^{1}H NMR (500 MHz, DMSO-d_6) δ 12.83 (s, 2H), 8.01 (d, J = 8.5 Hz, 2H), 7.58 (d, J = 8.5 Hz, 2H), 7.41 (d, J = 1.5 Hz, 1H), 7.20 (dd, J = 7.5, 1.5 Hz, 1H), 7.11 (d, J = 7.5 Hz, 1H), 5.17 (s, 2H).

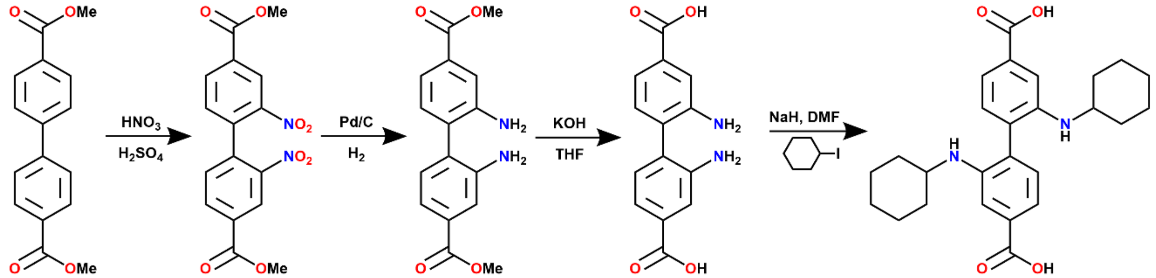

Figure S3. Synthesis of 2,2'-dicyclohexylamino-[1,1'-biphenyl]-4,4'-dicarboxylic acid.[2]

3.4. Dimethyl-2,2'-dinitro-[1,1'-biphenyl]-4,4'-dicarboxylate (1')

To a solution of commercially available dimethyl-biphenyl-4,4′-dicarboxylate (10 g, 37 mmol) in 100 mL of concentrated H_2SO_4 at 288-293 K, a mixture of nitric acid (56%, 12 mL, 74 mmol) in 15 mL of concentrated sulfuric acid was added dropwise. The reaction mixture was stirred vigorously for 2 h at 288-293 K and then was carefully poured onto ice (300 g). The pale yellow precipitated was filtered, washed with abundant cold water until reaching neutral pH, and air dried to obtain 10.5 g of product **1'** (yield: 85.7%). ^{1}H NMR (500 MHz, $CDCl_3$) δ: 8.90 (s, 2H), 8.37 (dd, J = 7.9, 1.7 Hz, 2H), 7.41 (d, J = 7.9 Hz, 2H), 4.03 (s, 6H).

3.5. Dimethyl 2,2'-diamino-[1,1'-biphenyl]-4,4'-dicarboxylate (2')

A 250-mL three-necked round-bottomed flask was charged with 3.2 g of 5% Pd/C, 5 g of 1' (13.8 mmol) and 165 mL of THF. Then, hydrogen was bubbled into the solution and the mixture was stirred overnight at room temperature under hydrogen atmosphere. After filtration over Celite, the solvent was removed *in vacuo*, affording 3.7g (90.6%) of product **2'** as a light-yellow solid. ^{1}H NMR (500 MHz, $CDCl_3$) δ: 7.52 – 7.045 (m, 4H), 7.17 (d, J = 7.8 Hz, 2H), 3.92 (s, 6H).

3.6. 2,2'-diamino-[1,1'-biphenyl]-4,4'-dicarboxylic acid (3')

Compound **2'** (3.75 g, 12.5 mmol) was dissolved in a mixture of 50:50 v/v THF/5% KOH (total volume 200 mL). The mixture was stirred overnight at 353 K. The aqueous layer was separated, then concentrated HCl was added until acidic pH was achieved to give a yellowish solid. The solid was filtered, washed with abundant cold water and air dried to obtain the desired product **3'** (3.33 g, 98%). ^{1}H NMR (400 MHz, DMSO-d_6) δ: 7.46 (d, J = 1.3 Hz, 2H), 7.27 (dd, J = 7.8, 1.3 Hz, 2H), 7.08 (d, J = 7.8 Hz, 2H).

3.7. 2,2'-dicyclohexylamino-[1,1'-biphenyl]-4,4'-dicarboxylic acid (4')

Compound **3'** (474 mg, 1.74 mmol) was dissolved in 2 mL of DMF in a 25 mL 3-neck flask under N_2 flow. NaH (192 mg, 8 mmol) was washed with hexane (2 mL, 3x) and dispersed

in 2 mL of DMF. The NaH dispersion was then added to 3-neck round-bottomed flask at 273 K. After stirring the mixture for 1 h, iodocyclohexane (840 mg, 4 mmol) was added dropwise. The reaction temperature was allowed to rise to room temperature, and the mixture was allowed to stir for 48 h under N_2 atmosphere. After completion of the reaction, the mixture was quenched with saturated $NaHCO_3$ solution. The resulting solution was washed with ethyl acetate (50 mL, 3x) to remove the unreacted iodocyclohexane. 1 M HCl was then added to the aqueous solution until reaching acidic pH to give a light brown solid (741 mg, 97.5%). ^1H NMR (400 MHz, DMSO-d_6) δ: 7.31 (s, J = 6.3 Hz, 2H), 7.27 (d, J = 7.8 Hz, 2H), 7.09 (d, J = 7.4 Hz, 2H), 1.75-1.99 (d, J =26.6 Hz, 4H), 1.47- 1.70 (s, 6H), 1.20-1.40 (s, 6H), 1.02-1.09 (d, J= 40.9 Hz, 6H).

4. Syntheses and characterization of MOFs

4.1. NH_2-UiO-67

A 0.4 M solution of $Zr(O^nPr)_4$ in CH_3COOH was prepared by mixing $Zr(O^nPr)_4$ 70 wt.% in n-propanol (187.2 mg, 0.4 mmol) with CH_3COOH (1 mL). A 0.02 M solution of H_2-NH_2-BPDC in DMF was prepared by dissolving H_2-NH_2-BPDC (15.4 mg, 0.06 mmol) in DMF (3 mL). To a 20 mL Pyrex vial were added in sequence CH_3COOH (0.6 mL), 0.4 M Zr $(O^nPr)_4$ solution (0.15 mL, 0.06 mmol), DMF (15 mL) and 0.02 M NH_2-BPDC solution (3 mL, 0.06 mmol). The vial was tightly capped and heated at 65 °C in an isothermal oven for 24 h to yield turbid yellow suspension. The suspension was centrifuged at 10000 rpm for 2 min to obtain yellow precipitate. The precipitate was washed with fresh DMF (16 mL, 4x) and dispersed in DMF (3 mL).

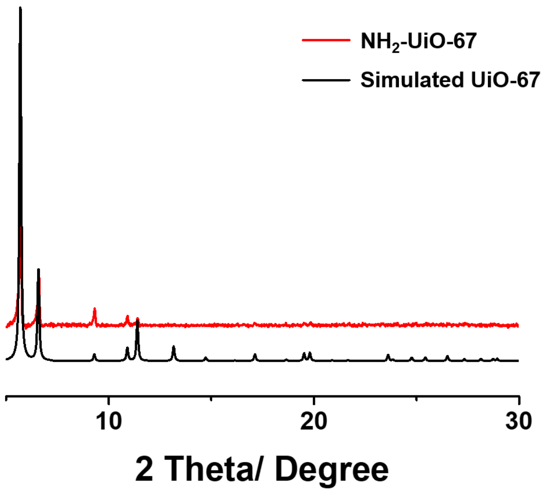

Figure S4. Simulated PXRD pattern of UiO-67 (black) and experimental PXRD pattern of as-synthesized NH_2-UiO-67 (red).

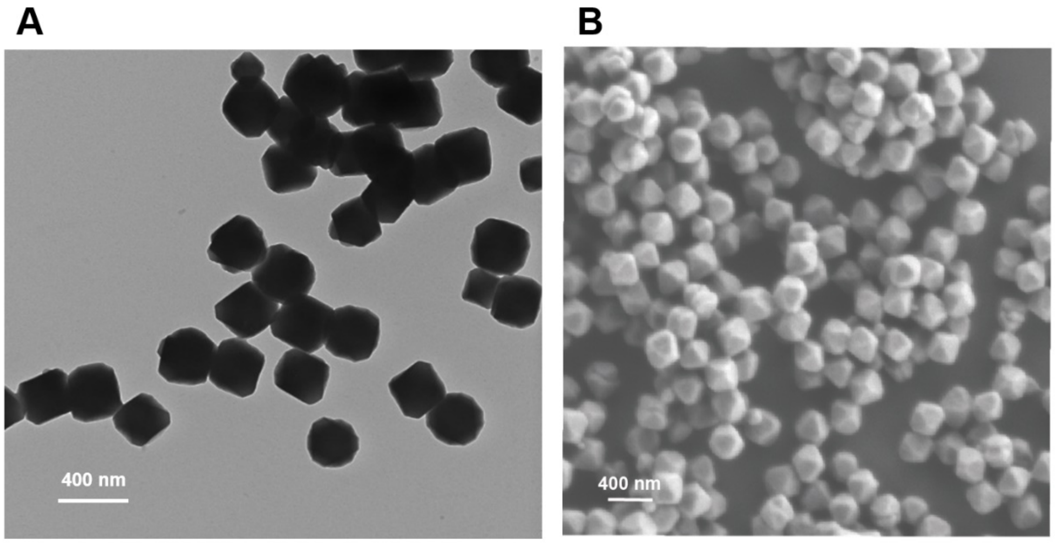

Figure S5. (A) TEM image of NH_2-UiO-67; (B) SEM image of NH_2-UiO-67.

4.2. $(CyNH)_2$-UiO-67

To a 20 mL Pyrex vial, 0.4 mmol $ZrCl_4$ (93 mg), 0.4 mmol H_2-$(CyNH)_2$-BPDC (174.5 mg) and 15 mL DMF were added. The mixture was sonicated for 5 minutes. Then, 2.76 mL CH_3COOH was added. The vial was tightly capped and heated at 100 °C in an isothermal oven for 16 h. The suspension was centrifuged at 10000 rpm for 2 min to obtain yellow precipitate. The precipitate was washed with fresh DMF (16 mL, 4x) and dispersed in DMF (3 mL).

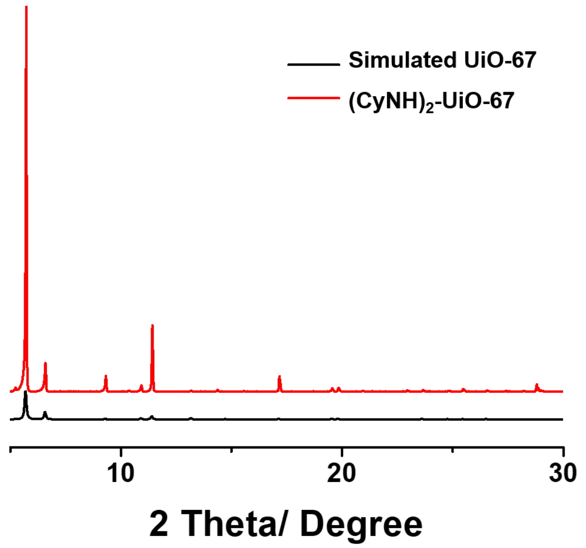

Figure S6. Simulated PXRD pattern of UiO-67 (black) and experimental PXRD pattern of as-synthesized $(CyNH)_2$-UiO-67 (red).

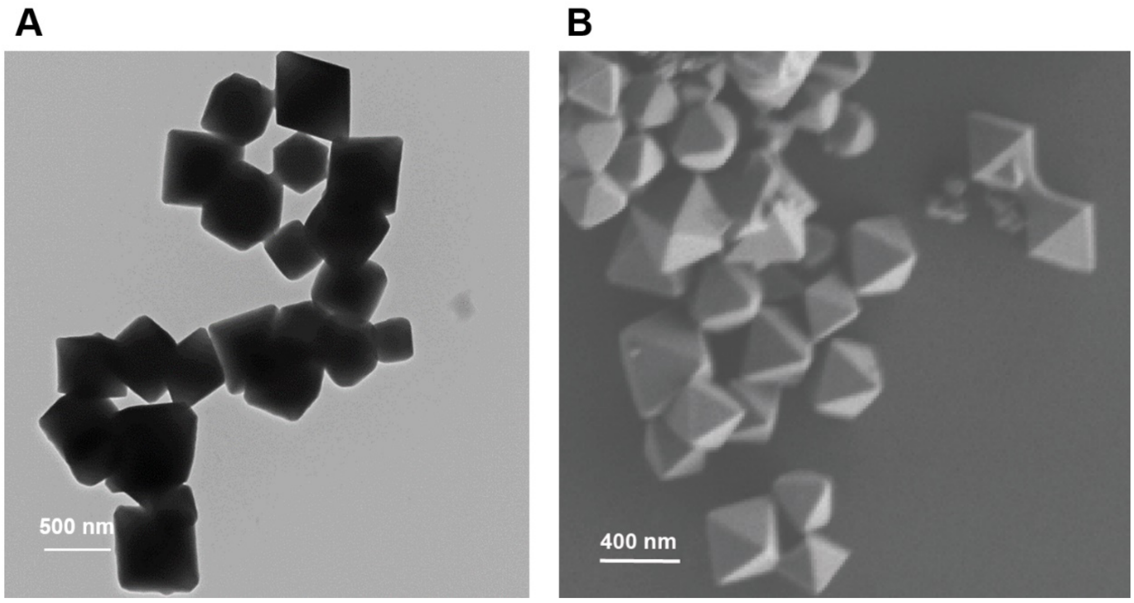

Figure S7. (A) TEM image of $(CyNH)_2$-UiO-67; (B) SEM image of $(CyNH)_2$-UiO-67.

4.3. $(CyNH)_2$-UiO-67⊂NH_2-UiO-67(cs-MOF-1)

$(CyNH)_2$-UiO-67 seeds were synthesized using the same method as $(CyNH)_2$-UiO-67 crystals (see above) but the reaction mixture was only heated for 6 h to afford smaller seed crystals. $(CyNH)_2$-UiO-67 seeds dispersed in 1 mL DMF were added to a 20 mL Pyrex vial. 0.4 mmol $ZrCl_4$ (93 mg), 0.4 mmol H_2-NH_2-BPDC (102 mg), 2.76 mL CH_3COOH and 15 mL DMF were then added. After sonicating for 5 min, the mixture was stirred with a magnetic stir bar (60 rpm) at 65 ºC. After 40 hours the reaction suspension was centrifuged at 10000 rpm for 2 min to obtain yellow precipitate product. The product was then washed with fresh DMF (4 mL, 4x).

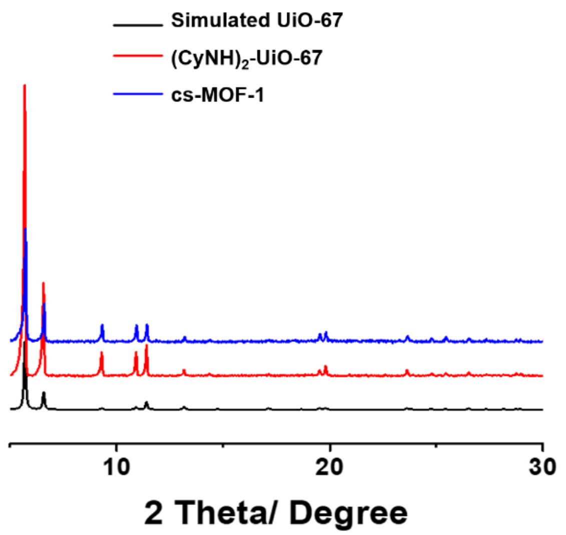

Figure S8. Simulated PXRD pattern of UiO-67 (black), experimental PXRD pattern of as-synthesized $(CyNH)_2$-UiO-67 seeds (red) and experimental PXRD pattern of as-synthesized cs-MOF-1 (blue).

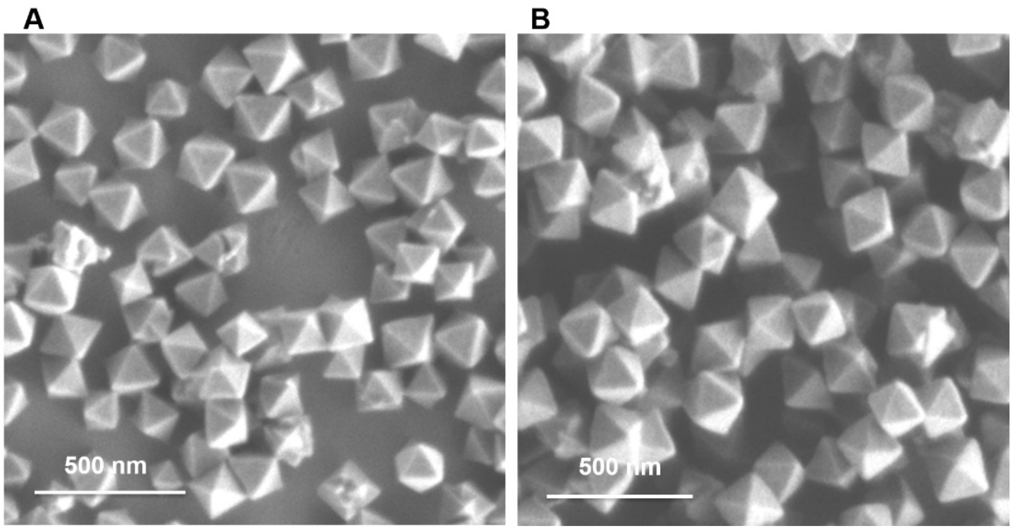

Figure S9. SEM image of $(CyNH)_2$-UiO-67 (A) and cs-MOF-1 (B).

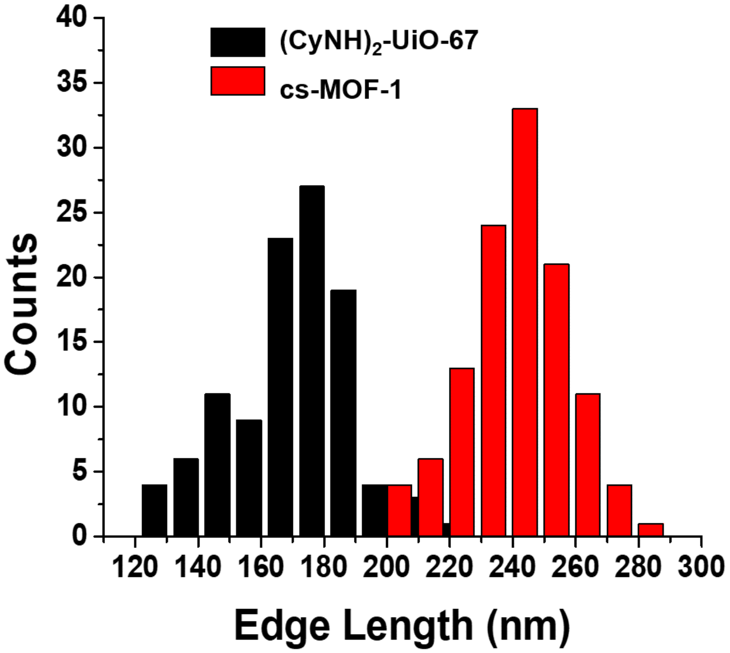

Figure S10. Size distributions (based on 100 counts) of $(CyNH)_2$-UiO-67 seeds with an average edge length of 174±36 nm (black) and cs-MOF-1 with an average edge length of 249±37 nm (red).

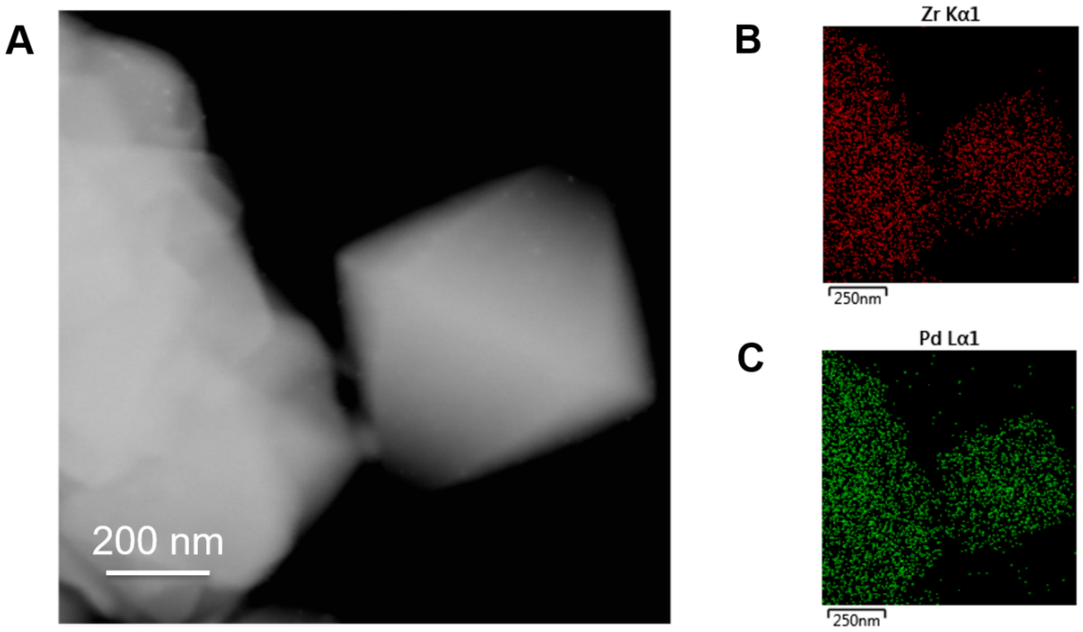

Figure S11. (A) STEM image of $(CyNH)_2$-UiO-67; (B) STEM-EDS mapping of Zr for $(CyNH)_2$-UiO-67; (C) STEM-EDS mapping of Pd for $(CyNH)_2$-UiO-67.

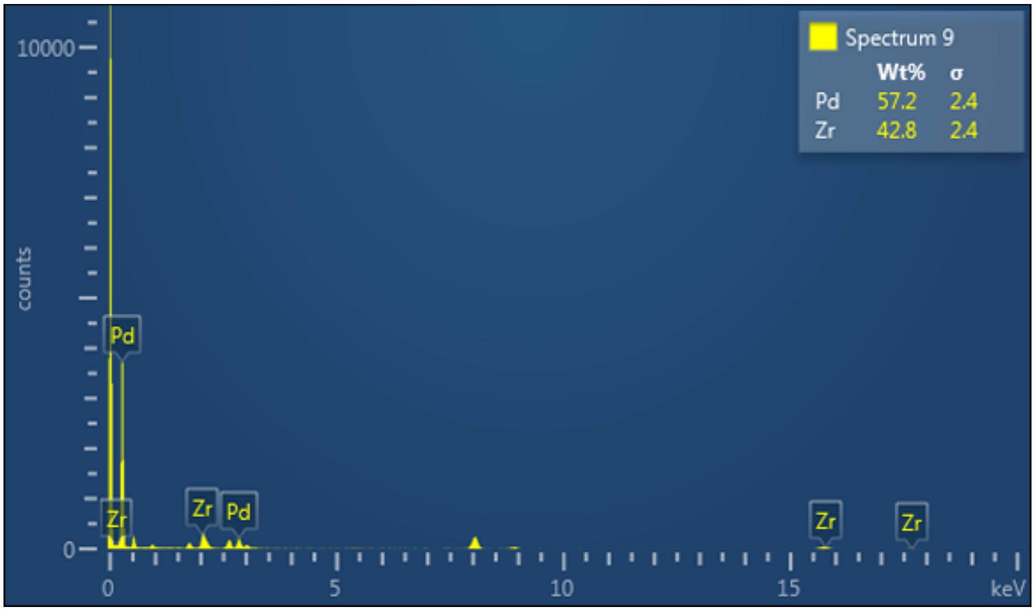

Figure S12. STEM-EDS point-scan result of Zr and Pd for $(CyNH)_2$-UiO-67.

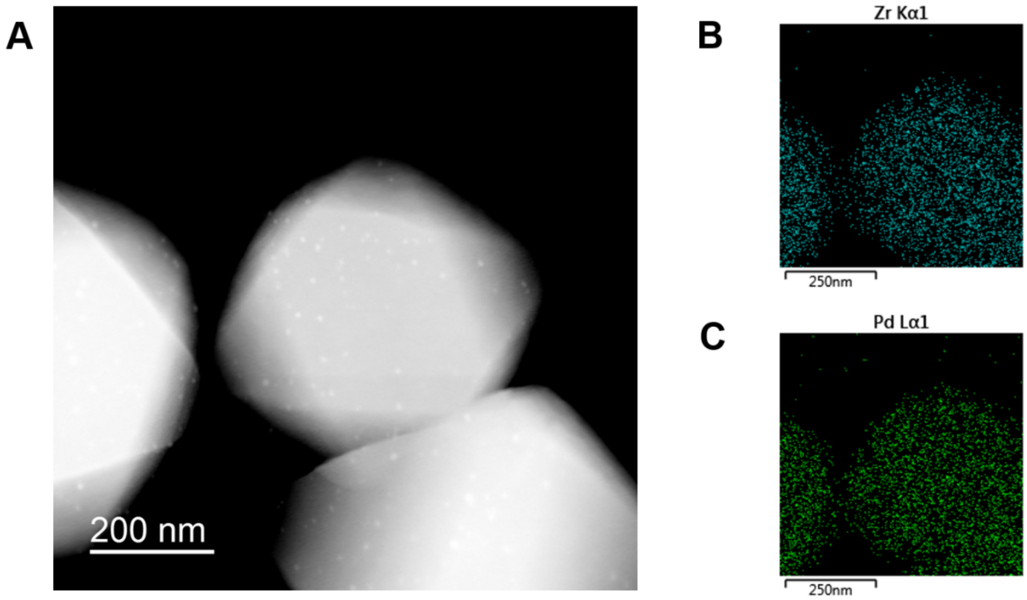

Figure S13. (A) STEM image of NH_2-UiO-67; (B) STEM-EDS mapping of Zr for NH_2-UiO-67; (C) STEM-EDS mapping of Pd for NH_2-UiO-67.

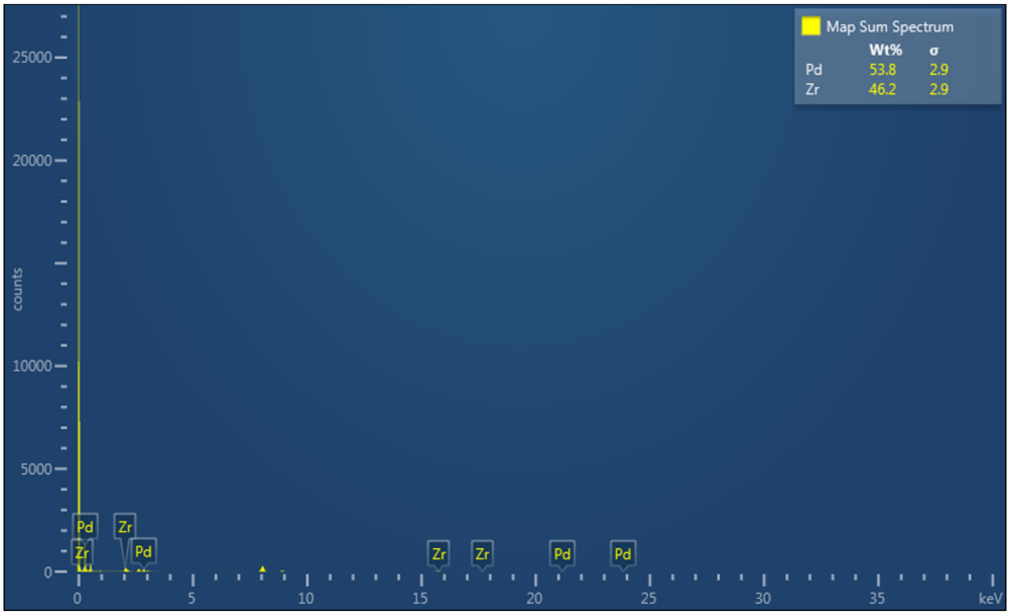

Figure S14. STEM-EDS point-scan result of Zr and Pd for NH_2-UiO-67.

4.4. NH_2-UiO-67⊂$(CyNH)_2$-UiO-67 (cs-MOF-2)

NH_2-UiO-67 seeds dispersed in 1 mL DMF were added to a 20 mL Pyrex vial. 0.4 mmol $ZrCl_4$ (93 mg), 0.4 mmol H_2-$(CyNH)_2$-BPDC (174.5 mg), 2.76 mL CH_3COOH and 15 mL DMF were then added. The mixture was vortexed and incubated at 120 ºC in an isothermal oven for 24 h. The reaction suspension was centrifuged at 10000 rpm for 2 min to obtain yellow precipitate product. The product was then washed with fresh DMF (4 mL, 4x).

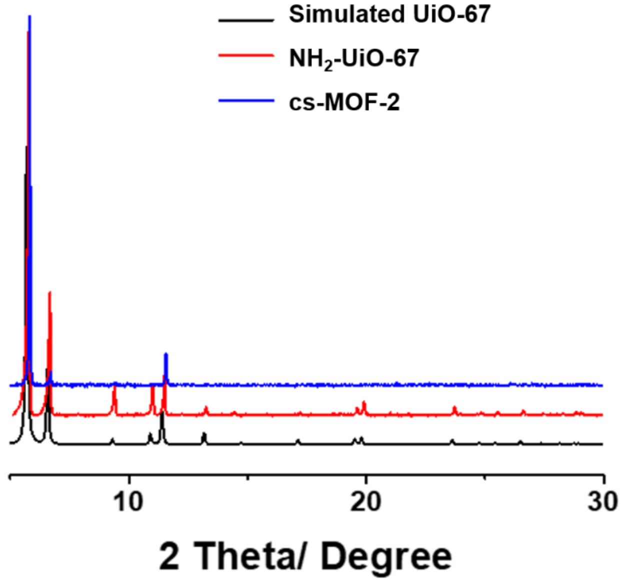

Figure S15. Simulated PXRD pattern of UiO-67 (black), experimental PXRD pattern of as-synthesized NH_2-UiO-67 seeds (red) and experimental PXRD pattern of as-synthesized cs-MOF-2 (blue).

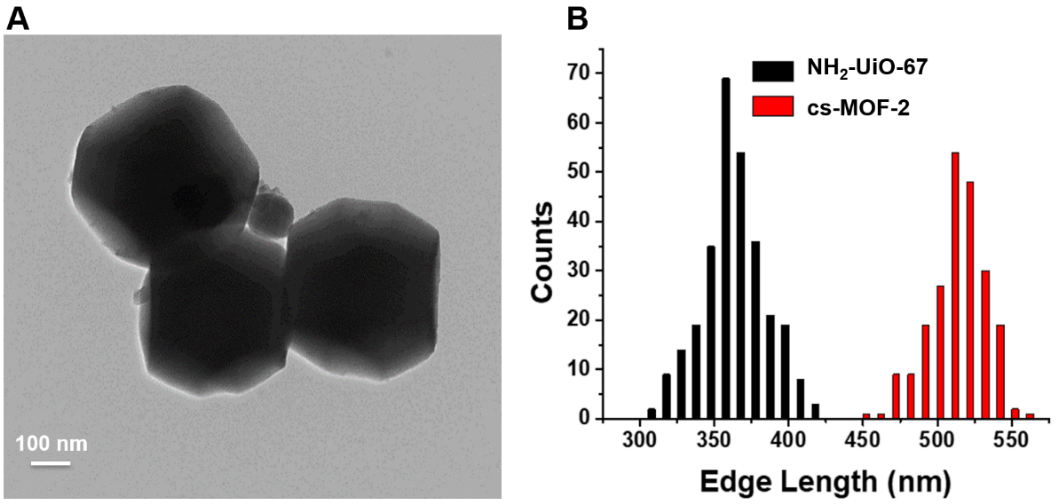

Figure S16. (A) TEM image of cs-MOF-2; (B) Size distributions (based on 300 counts) of NH_2-UiO-67 seeds with an average edge length of 363±22 nm (black) and cs-MOF-2 with an average edge length of 506±31 nm (red).

4.5. $(CyNH)_2$-UiO-67⊂UiO-67

$(CyNH)_2$-UiO-67 seeds were synthesized using the same method as $(CyNH)_2$-UiO-67 crystals (see above) but the reaction mixture was only heated for 6 h to afford smaller seed crystals. $(CyNH)_2$-UiO-67 seeds dispersed in 1 mL DMF were added to a 20 mL Pyrex vial. 0.4 mmol $ZrCl_4$ (93 mg), 0.4 mmol H_2-BPDC (96.9 mg), 1.08 mL CH_3COOH and 15 mL DMF were then added. After sonicating for 5min, the mixture was stirred with a magnetic stir bar (60 rpm) at 65 °C. After 24 hours the reaction suspension was centrifuged at 10000 rpm for 2 min to obtain yellow precipitate product. The product was then washed with fresh DMF (4 mL, 4x).

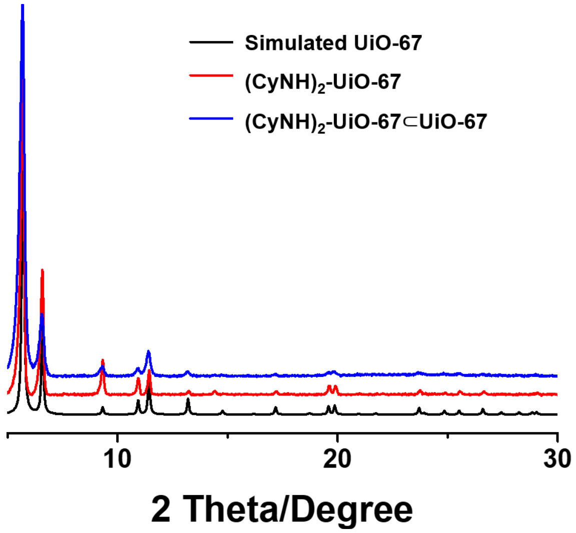

Figure S17. Simulated PXRD pattern of UiO-67 (black), experimental PXRD pattern of as-synthesized $(CyNH)_2$-UiO-67 seeds (red) and experimental PXRD pattern of as-synthesized $(CyNH)_2$-UiO-67⊂UiO-67 (blue).

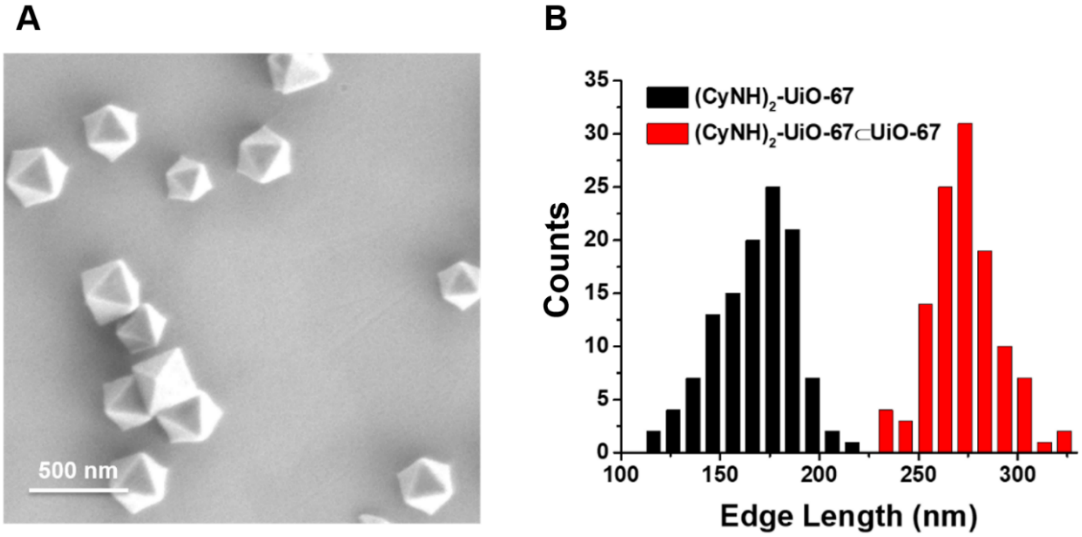

Figure S18. (A) SEM image of $(CyNH)_2$-UiO-67⊂UiO-67; (B) Size distributions (based on 300 counts) of $(CyNH)_2$-UiO-67 seeds with an average edge length of 171±37 nm (black) and $(CyNH)_2$-UiO-67⊂UiO-67 with an average edge length of 270±32 nm (red).

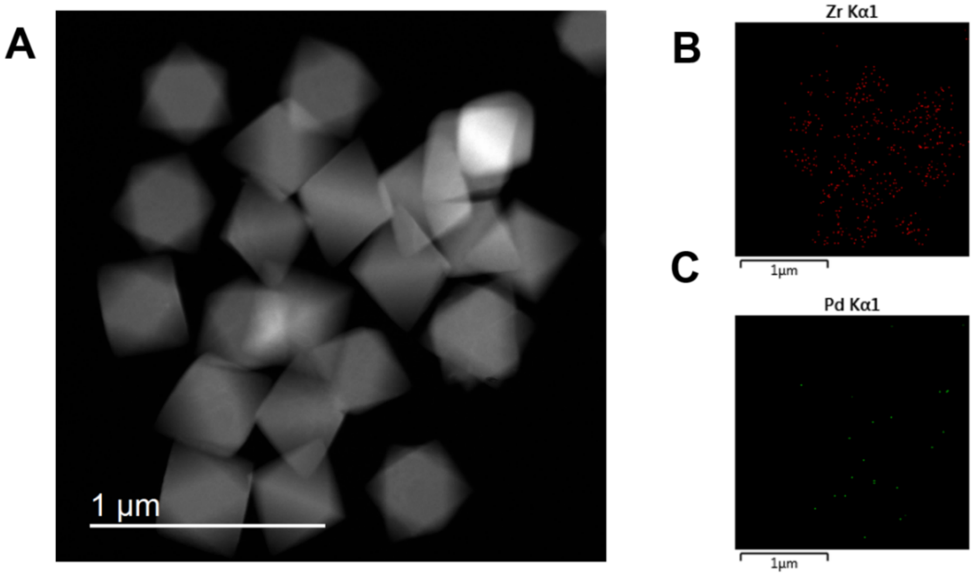

Figure S19. (A) STEM image of UiO-67; (B) STEM-EDS mapping of Zr for UiO-67; (C) STEM-EDS mapping of Pd for UiO-67.

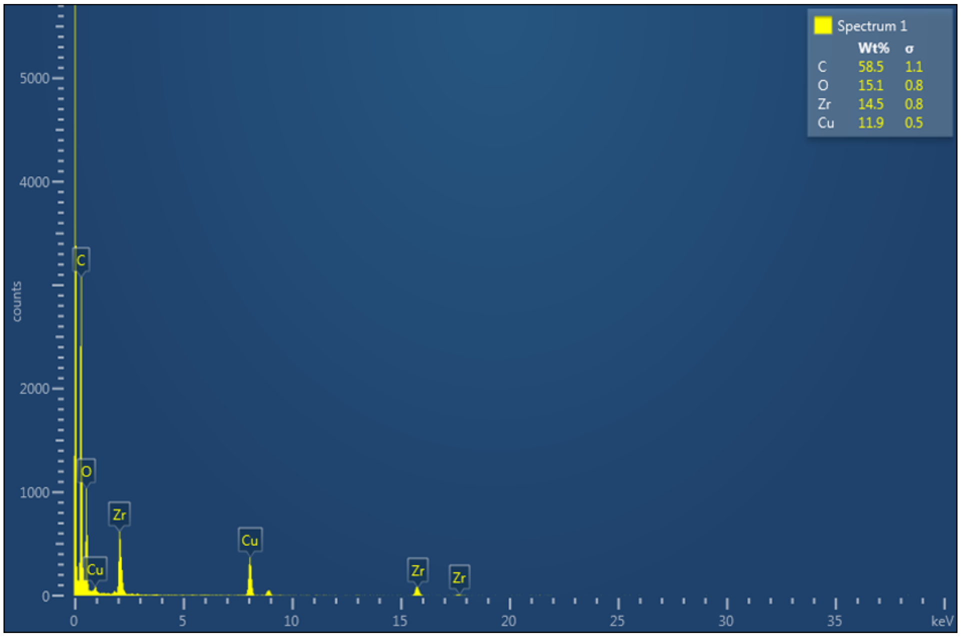

Figure S20. STEM-EDS point-scan results for UiO-67.

5. Single-gas adsorption isotherms

5.1. N_2 adsorption isotherms at 77 K

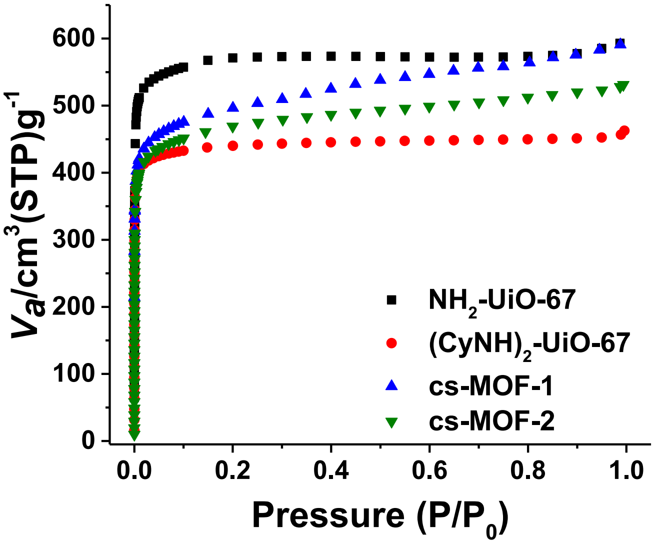

Figure S21. N_2 adsorption isotherms at 77 K of NH_2-UiO-67 (black square), $(CyNH)_2$-UiO-67 (red circle), cs-MOF-1 (blue up triangle), and cs-MOF-2 (green down triangle). The calculated BET surface areas were 2280 m^2g^{-1} (NH_2-UiO-67), 1780 m^2g^{-1} ($(CyNH)_2$-UiO-67), 1830 m^2g^{-1} (cs-MOF-1) and 1810 m^2g^{-1} (cs-MOF-2).

5.2. CO_2 adsorption isotherms at 298 K

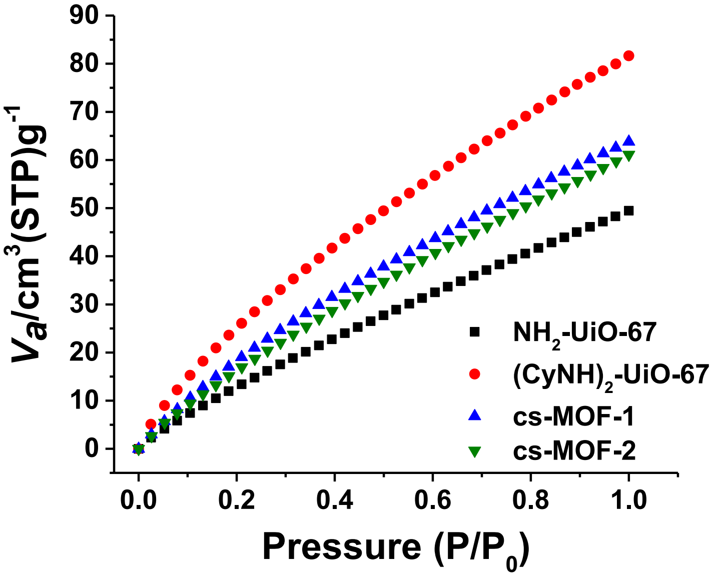

Figure S22. CO_2 adsorption isotherms at 298 K of NH_2-UiO-67 (black square), $(CyNH)_2$-UiO-67 (red circle), cs-MOF-1 (blue up triangle), and cs-MOF-2 (green down triangle).

5.3. N_2 adsorption isotherms at 298 K

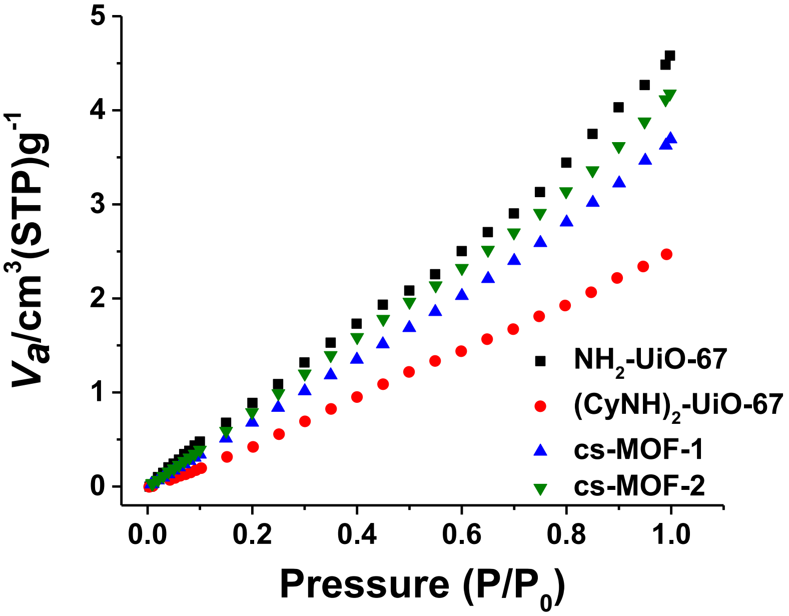

Figure S23. N_2 adsorption isotherms at 298 K of NH_2-UiO-67 (black square), $(CyNH)_2$-UiO-67 (red circle), cs-MOF-1 (blue up triangle), and cs-MOF-2 (green down triangle).

6. Multi-gas testing

6.1. System design

A multi-gas test system was built based on literature.[3]

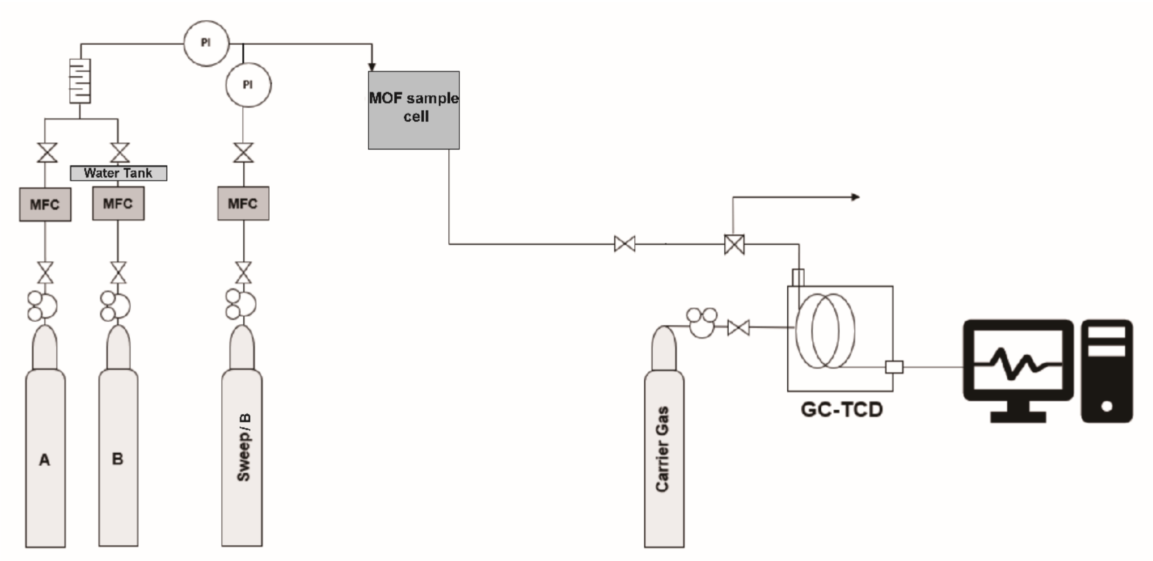

Scheme S1. Schematic diagram of multi-gas system (A, B: CO_2, N_2; MFC: mass flow controller; PI: pressure indicator).

6.2. Multi-gas tests results

After solvent exchange using the same method as gas adsorption isotherm tests, the MOF samples were activated at 120 °C for 24 hours in a vacuum oven before multi-gas test in order to remove any solvent molecules potentially remaining in the pore. For multi-gas measurement, ~100 mg of sample was sealed in a gas tight module with silicon o-rings. The feed gas was purged through the module for 12 h (Scheme S1). The feed contained a mixture of N_2 and CO_2 (85/15) with 0%, 15% and 30% relative humidity with a total feed flow rate of 200 mL/min; feed composition was adjusted using mass flow controller. The MOF sample was then re-activated at 120 °C. The compositions of desorbed gas were analyzed by gas chromatography (Agilent-GC8860). He gas was introduced with volumetric flow rate of 50 mL/min as a sweep gas. The plot of velocity-time was used to calculate the uptake of each gas. Tests at each condition were performed twice to confirm the reliability of the results and the average gas uptake of two trials was used for analysis. After multi-gas test at each condition, PXRD patterns and N_2 adsorption isotherms at 77 K were collected to assess the integrity of the MOFs.

Table S1. Multi-gas tests results at 0% RH.

	CO_2 uptake at 0% RH (cc/g)	N_2 uptake at 0% RH (cc/g)	CO_2/N_2 adsorption selectivity
**NH_2-UiO-67**	2.02	1.25	9.16
**$(CyNH)_2$-UiO-67**	6.53	1.56	23.7
**cs-MOF-1**	2.31	1.22	10.7
**cs-MOF-2**	3.24	1.24	14.8

Table S2. Multi-gas tests results at 15% RH.

	CO_2 uptake at 15% RH (cc/g)	N_2 uptake at 15% RH (cc/g)	CO_2/N_2 adsorption selectivity	CO_2 uptake decrease under humidity	Selectivity decrease under humidity
**NH_2-UiO-67**	1.62	1.24	7.40	20%	19%
**$(CyNH)_2$-UiO-67**	2.86	1.51	10.7	56%	55%
**cs-MOF-1**	2.23	1.34	9.43	3.7%	12%
**cs-MOF-2**	1.96	1.28	8.68	40%	41%

Table S3. Multi-gas tests results at 30% RH.

	CO_2 uptake at 30% RH (cc/g)	N_2 uptake at 30% RH (cc/g)	CO_2/N_2 adsorption selectivity	CO_2 uptake decrease under humidity	Selectivity decrease under humidity
**NH_2-UiO-67**	1.38	1.19	6.57	32%	28%
**$(CyNH)_2$-UiO-67**	2.50	1.55	9.14	62%	61%
**cs-MOF-1**	1.82	1.35	7.64	21%	28%
**cs-MOF-2**	1.59	1.32	6.83	51%	54%

6.3. Characterization of MOF stability after Multi-gas Tests

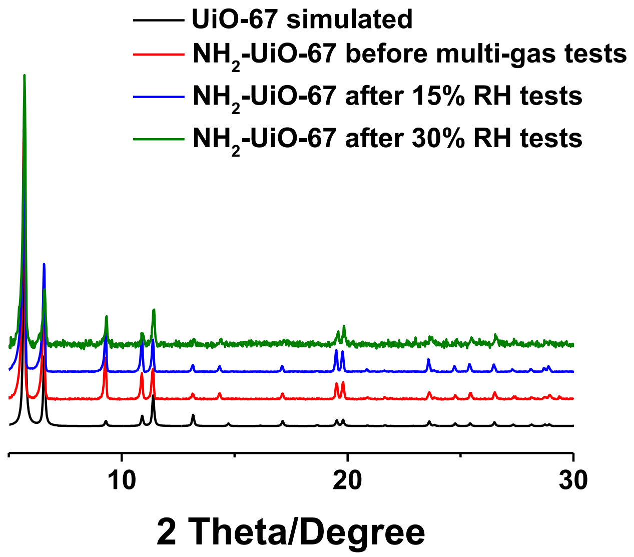

Figure S24. Simulated PXRD pattern of UiO-67 (black), experimental PXRD pattern of NH_2-UiO-67 before multi-gas tests (red), NH_2-UiO-67 after 15% RH tests (blue) and NH_2-UiO-67 after 30% RH tests (green).

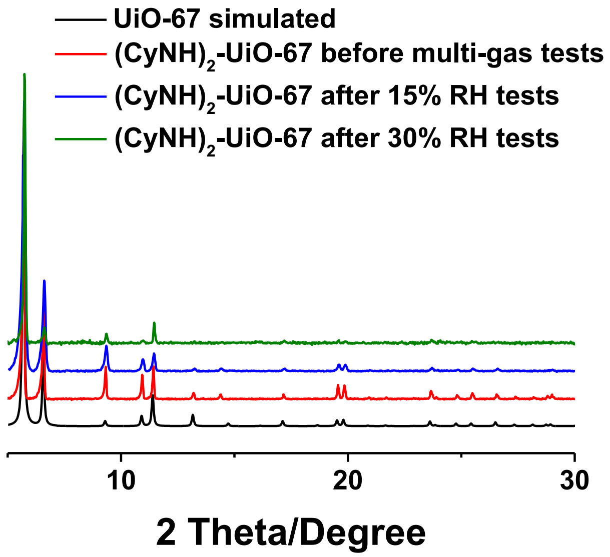

Figure S25. Simulated PXRD pattern of UiO-67 (black), experimental PXRD pattern of $(CyNH)_2$-UiO-67 before multi-gas tests (red), $(CyNH)_2$-UiO-67 after 15% RH tests (blue) and $(CyNH)_2$-UiO-67 after 30% RH tests (green).

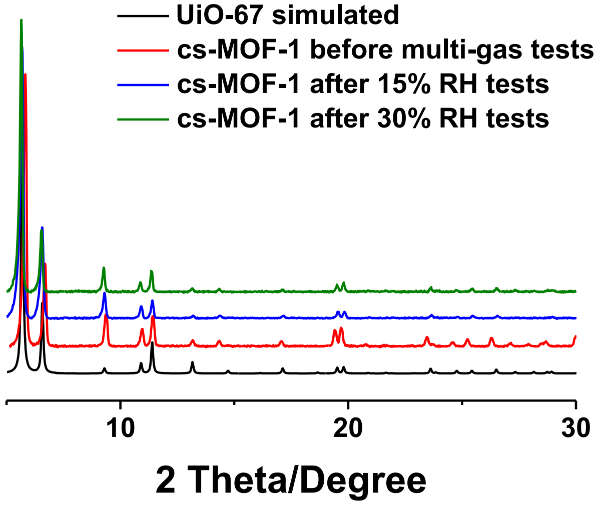

Figure S26. Simulated PXRD pattern of UiO-67 (black), experimental PXRD pattern of cs-MOF-1 before multi-gas tests (red), cs-MOF-1 after 15% RH tests (blue) and cs-MOF-1 after 30% RH tests (green).

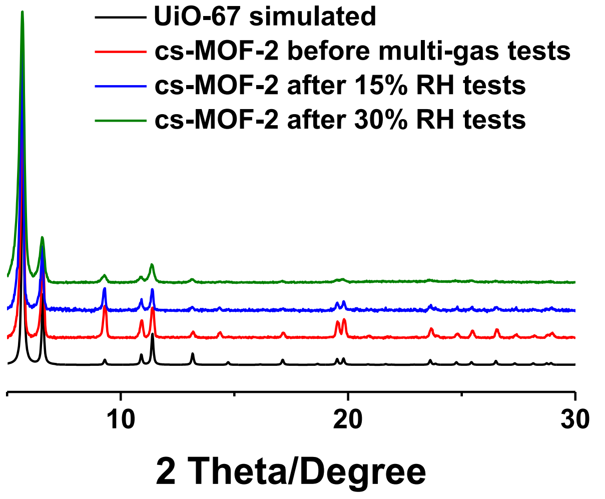

Figure S27. Simulated PXRD pattern of UiO-67 (black), experimental PXRD pattern of cs-MOF-2 before multi-gas tests (red), cs-MOF-2 after 15% RH tests (blue) and cs-MOF-2 after 30% RH tests (green).

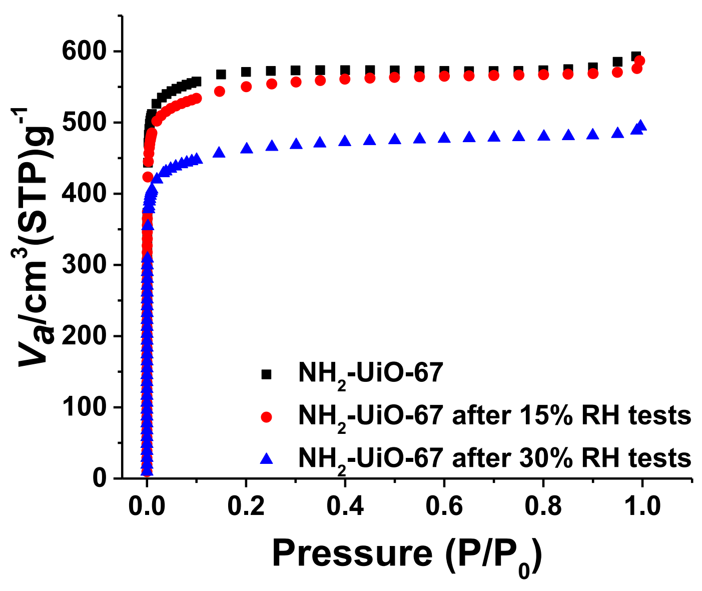

Figure S28. N_2 adsorption isotherms at 77 K of NH_2-UiO-67 before multi-gas tests (black square), after 15% RH tests (red circle) and after 30% RH tests (blue triangle). The calculated BET surface areas were 2280 m^2g^{-1} (before multi-gas tests), 2150 m^2g^{-1} (after 15% RH tests) and 1810 m^2g^{-1} (after 30% RH tests).

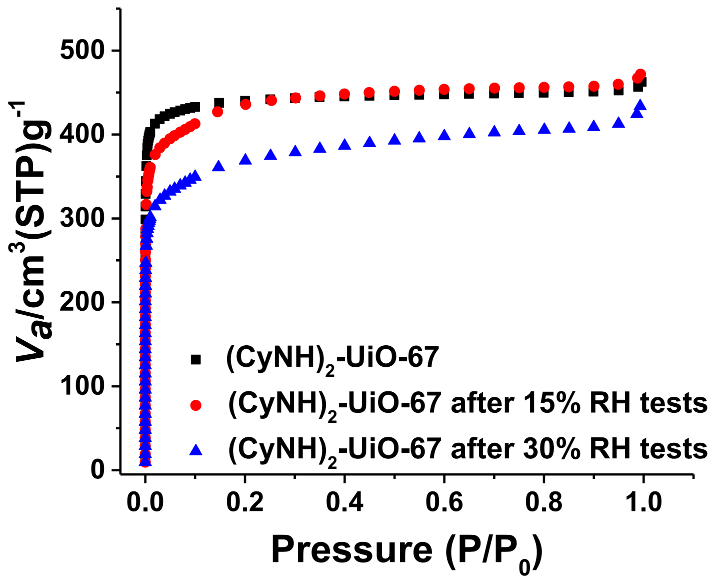

Figure S29. N_2 adsorption isotherms at 77 K of $(CyNH)_2$-UiO-67 before multi-gas tests (black square), after 15% RH tests (red circle) and after 30% RH tests (blue triangle). The calculated BET surface areas were 1780 m^2g^{-1} (before multi-gas tests), 1650 m^2g^{-1} (after 15% RH tests) and 1390 m^2g^{-1} (after 30% RH tests).

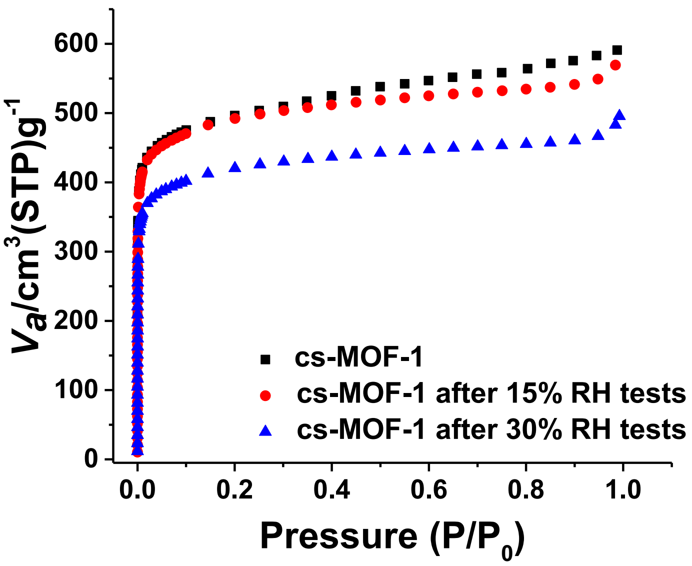

Figure S30. N_2 adsorption isotherms at 77 K of cs-MOF-1 before multi-gas tests (black square), after 15% RH tests (red circle) and after 30% RH tests (blue triangle). The calculated BET surface areas were 1830 m^2g^{-1} (before multi-gas tests), 1820 m^2g^{-1} (after 15% RH tests) and 1610 m^2g^{-1} (after 30% RH tests).

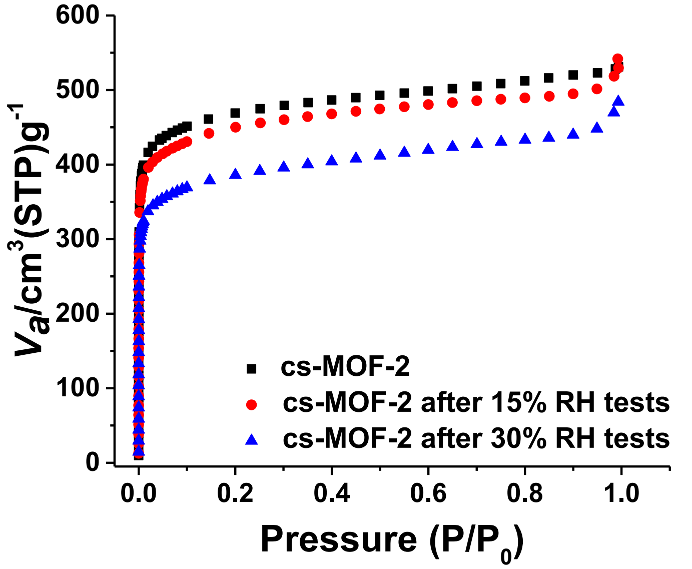

Figure S31. N_2 adsorption isotherms at 77 K of cs-MOF-2 before multi-gas tests (black square), after 15% RH tests (red circle) and after 30% RH tests (blue triangle). The calculated BET surface areas were 1810 m^2g^{-1} (before multi-gas tests), 1730 m^2g^{-1} (after 15% RH tests) and 1480 m^2g^{-1} (after 30% RH tests).

7. References

(1) Liu, C.; Li, T.; Rosi, N. L. Strain-Promoted “Click” Modification of a Mesoporous Metal–Organic Framework. *J. Am. Chem. Soc.* **2012**, *134*, 18886, DOI: 10.1021/ja307713q

(2) Gil-San-Millan, R.; Lopez-Maya, E.; Hall, M.; Padial, N. M.; Peterson, G. W.; DeCoste, J. B.; Rodriguez-Albelo, L.M.; Oltra, J. E.; Barea, E.; Navarro, J. A. R. Chemical Warfare Agents Detoxification Properties of Zirconium Metal–Organic Frameworks by Synergistic Incorporation of Nucleophilic and Basic Sites. *ACS Appl. Mater. Interfaces* **2017**, *9*, 28, 23967, DOI: 10.1021/acsami.7b06341

(3) Kumar, A.; Madden, D. G.; Lusi, M.; Chen, K-J.; Daniels, E. A.; Curtin, T.; Perry IV, J. J.; Zaworotko, M. J. Direct Air Capture of CO_2 by Physisorbent Materials. *Angew. Chem. Int. Ed.* **2015,** *54*, 14372, DOI: 10.1002/anie.201506952
